# O.6.3-9 Intervention adaption protocol for the holistic school-based intervention, Youth-Physical Activity Towards Health, within a Danish context

**DOI:** 10.1093/eurpub/ckad133.289

**Published:** 2023-09-11

**Authors:** Thea Toft Amholt, Mette Kurtzhals, Paulina Melby, Peter Elsborg, Glen Nielsen, Peter Bentsen

**Affiliations:** Center for Clinical Research and Prevention, Bispebjerg and Frederiksberg Hospital; Department of Nutrition, Exercise and Sports, University of Copenhagen, Denmark; Department of Sports Science and Clinical Biomechanics, University of Southern Denmark; thea.toft.amholt@regionh.dk; Center for Clinical Research and Prevention, Bispebjerg and Frederiksberg Hospital; Department of Nutrition, Exercise and Sports, University of Copenhagen, Denmark; Center for Clinical Research and Prevention, Bispebjerg and Frederiksberg Hospital

## Abstract

**Introduction:**

In recent years, the concept of physical literacy (PL) has gained interest worldwide. Yet, only a few interventions targeting PL exist on a global scale. A promising theory-based and nationally tested intervention, Youth-Physical Activity Towards Health (Y-PATH), has proven to be effective in improving children's and adolescents’ physical activity levels and motor skills in Ireland. Using this protocol, we aim to context-adapt the Irish Y-PATH into an intervention that works through the schools in Denmark and targets PL among pupils in 4th and 5th grades.

**Methods and analysis:**

We apply a stepwise approach to intervention adaptation, inspired by the Medical Research Council’s guidance for developing complex interventions as well as the ‘consensus informed guidance for adapting interventions to achieve a good fit between the intervention and context’ (ADAPT) process model and its principles and actions. First, we combine evidence synthesis with stakeholders' and researchers’ engagement to identify relevant and tentative adaptations and to understand barriers and enablers of the promotion of physical literacy in school-based interventions. Next, we adjust and improve our intervention adaptation ideas by collecting detailed data through interviews with purposively sampled stakeholders and pre-pilot tests of selected prototypes of materials in a ‘real-world’ setting. Finally, we clarify the intervention adaptations – its components, materials, and content – in a discussion with purposively sampled stakeholders, and finally, we test the study design.

**Expected results and outcomes:**

A Danish version of the Y-PATH intervention, ready for feasibility testing within a Danish context, and a TIDieR intervention description checklist suitable for testing and further refinement in a feasibility study. The process will further increase our knowledge and understanding of the Danish school context as well as the teacher and pupils’ needs for an intervention promoting PL. In this presentation, we will present the preliminary results from the adaptation process, including the intervention components, materials, and content.

**Funding:**

The intervention adaptation forms part of an overarching feasibility study that is financed by the Foundation TrygFonden (Grant ID #153181).

